# Effectiveness of electronic patient-reported outcomes in patients with cancer undergoing immunotherapy: a real-world retrospective study

**DOI:** 10.1007/s10147-026-03032-z

**Published:** 2026-04-13

**Authors:** Hideyuki Katsura, Yukio Suga, Shinji Kimoto, Reiko Ando Makihara, Hiroshi Ota, Hitomi Toi, Naoko Takata, Hiroaki Ikesue

**Affiliations:** 1 Department of Pharmacy, Komatsu Municipal Hospital, HO-60, Mukaimotoori-machi, Komatsu, Ishikawa 923-8560 Japan; 2 Department of Clinical Drug Informatics, Faculty of Pharmacy, Institute of Medical, Pharmaceutical and Health Science, Kanazawa University, Kanazawa, Ishikawa Japan; 3 Department of Pharmacy, Takeda General Hospital, Aizuwakamatsu, Fukushima Japan; 4 Department of Pharmacy, National Cancer Center Hospital, Tokyo, Japan; 5 Department of Pharmacy, Nagoya University Hospital, Nagoya, Aichi Japan

**Keywords:** Electronic patient-reported outcome, Immune checkpoint inhibitors, Immune-related adverse events

## Abstract

**Background:**

Immune checkpoint inhibitors (ICIs) are essential in advanced cancer treatments; however, they can cause severe immune-related adverse events (irAEs). Herein, we aimed to investigate the effectiveness of electronic patient-reported outcomes (ePROs) in monitoring the symptoms of patients with advanced cancer undergoing ICI therapy.

**Methods:**

We retrospectively reviewed the medical records of patients with advanced cancer who received ICIs at the Komatsu Municipal Hospital between June 2019 and March 2024 following ePROs introduction.

**Results:**

Overall, 168 patients were included, with 43 and 125 in the ePRO and conventional care groups, respectively. The incidence of grade ≥ 3 irAEs was significantly lower in the ePRO than the conventional care group (7% vs. 21%; P = 0.039). Multivariate analysis showed that ePROs use was significantly associated with fewer emergency visits (odds ratio [OR]: 0.22, P < 0.001) and hospitalizations (OR: 0.36, P = 0.027). Patients in the ePRO group demonstrated significantly longer progression-free survival (PFS) and overall survival (OS) than did those in the conventional care group (median PFS: 10.8 vs. 4.8 months, hazard ratio [HR]: 0.50, P = 0.003; median OS: not reached vs. 17.0 months, HR: 0.40, P = 0.010). These survival benefits remained statistically significant after propensity score matching (n = 37 per group; PFS: HR 0.48, P = 0.009; OS: HR 0.37, P = 0.013).

**Conclusion:**

Implementation of ePROs was associated with clinical benefits in patients with advanced cancer receiving ICI therapy. ePRO-based symptom monitoring may contribute to safer and more effective immunotherapy in real-world oncology practice.

**Supplementary Information:**

The online version contains supplementary material available at 10.1007/s10147-026-03032-z.

## Introduction

The advent of immune checkpoint inhibitors (ICIs) as first- and second-line therapies has revolutionized treatment for advanced cancer, offering the potential for long-term remission and improved survival [1, 2]. However, ICIs are associated with immune-related adverse events (irAEs), some life-threatening, with a 7.3% mortality rate among affected patients [3]. The management of irAEs often relies on trial and error [4], underscoring the need for careful monitoring and early detection.

Electronic patient-reported outcomes (ePROs) are a recommended tool in clinical practice for managing symptoms in patients with advanced cancers, ensuring that no symptoms are overlooked [5]. ePROs enhance patient–provider communication and have been shown to reduce emergency visits and hospitalizations while improving treatment outcomes [6–8]. In particular, for patients receiving immune checkpoint inhibitors (ICIs), symptom onset is often unpredictable and sometimes less perceptible to patients, making continuous monitoring important for early recognition and timely intervention. The ePRO follow-up model can reduce the incidence of grade 3–4 irAEs and emergency department presentations in patients receiving ICIs [9]. However, the effectiveness of ePRO-guided approaches in patients receiving ICI-based chemotherapy—particularly regarding survival benefits—remains debated, and further real-world evidence is needed to clarify its impact.

ePRO-based symptom monitoring has been shown feasible and acceptable in various oncology settings, including home-based and postoperative contexts [10, 11]. Here, we retrospectively evaluated whether ePRO use reduces emergency visits, hospitalizations, progression-free survival (PFS), and overall survival (OS) in patients with advanced cancer receiving ICIs.

## Patients and methods

### Study participants

We conducted a retrospective review of patients’ medical records. Data were collected from consecutive patients who received ICI or ICI-based chemotherapy between June 2019—when the ePROs were first introduced—and March 2024 at Komatsu Municipal Hospital. The administered ICIs included nivolumab, pembrolizumab, atezolizumab, and durvalumab. All patients scheduled to initiate ICI therapy with or without chemotherapy were asked about their willingness to use ePROs. Patients who started ICIs at other hospitals, received ICIs as perioperative treatment, or were undergoing third-line or later treatments, were excluded.

###  Ethical approval

Ethical approval and waiver of informed consent were obtained from the Institutional Review Board of Komatsu Municipal Hospital (no. 02–23).

### ePRO system

This study utilized the ePRO MedicalCare Station^®^ [12], Welby My Carte ONC^®^ [13], and 3H P-Guardian^®^ [14]. Pharmacists conducted follow-up using these platforms; no provider received formal training on the applications or process. All three platforms assessed symptoms using PRO-CTCAE-aligned severity grading with assessments scheduled at least weekly; Welby MyKarte ONC^®^ and MedicalCare Station^®^ also offered a face-scale option, and MedicalCare Station^®^ used simplified, patient-friendly symptom descriptions with a 4-point severity scale. Although interfaces differed, all platforms included the following core irAE-relevant symptoms: fatigue, fever, rash, dyspnea, diarrhea, loss of appetite, nausea, shortness of breath, cough, edema, palpitations, headache, and myalgia.

All platforms enabled remote symptom self-reporting from home, with daily entry available. Before initiating ICI therapy, a pharmacist provided each patient with a structured explanation using a dedicated pamphlet and guided them through their first entry session. Automated reminders were sent to non-responders, and patients were instructed to report at least once per week. If grade ≥ 3 AEs were detected, standardized guidance was automatically delivered to both patients and pharmacists. Pharmacists reviewed entries at least once each weekday, and all patient-reported data were summarized and reported to the attending physician via the electronic medical record system. Scheduled telephone follow-up was not conducted in the ePRO group; telephone contact was initiated only when warranted by application-reported data. If severe AEs were suspected, patients were advised to visit the hospital. In the conventional care group, symptom assessment was performed by physicians and nurses at scheduled clinic visits, with no systematic mechanism for capturing inter-visit symptom changes unless patients independently contacted the hospital.

### Data collection

The following data were collected: age, sex, Eastern Cooperative Oncology Group performance status (PS), cancer type, body surface area, neutrophil-to-lymphocyte ratio (NLR), number of prior treatment lines, chemotherapy regimen, smartphone ownership, emergency visits, hospitalizations, PFS, and OS. The survival of patients who received ICI-based chemotherapy was defined as the time from treatment initiation to death from any cause or the last follow-up (March 31, 2024). PFS was defined as the time from the initiation of ICI-based chemotherapy to objective evidence of tumor progression (as determined by the Response Evaluation Criteria in Solid Tumors [RECIST], version 1.1), death from any cause, or last follow-up, whichever occurred first. The response to ICI-based chemotherapy was assessed using RECIST, version 1.1. The safety profile was assessed and graded using the Common Terminology Criteria for Adverse Events, version 5.0.

### Statistical analysis

Categorical data are expressed as frequencies and percentages and compared between the groups using the chi-square test. Continuous data are expressed as medians (interquartile ranges [IQRs]) and were compared using the Mann–Whitney U test. Odds ratios (ORs) and 95% confidence intervals (CIs) were calculated using univariate and multivariate logistic regression analyses to evaluate the factors associated with emergency visits or hospitalizations. Hazard ratios (HRs) and 95% CIs were calculated using univariate and multivariate Cox proportional hazard models to assess the factors affecting PFS or OS. OS and PFS were estimated using the Kaplan–Meier method, and between-group differences were assessed using the log-rank test. Propensity scores were estimated using a logistic regression model including cancer type (NSCLC vs. non-NSCLC), ECOG PS, age, and smartphone ownership, with one-to-one nearest-neighbor matching without replacement using a caliper of 0.2 standard deviations of the logit. Balance was confirmed by comparing baseline characteristics before and after matching, and PFS and OS were estimated using the Kaplan–Meier method in the matched cohort. Propensity score matching was performed using EZR version 1.61 (Saitama Medical Center, Jichi Medical University, Saitama, Japan); all other analyses were performed using IBM SPSS Statistics version 24.0 (IBM Corp., Armonk, NY, USA). The primary endpoints were the incidence of grade ≥ 3 irAEs and emergency visits. Secondary endpoints included the hospitalization rate, PFS, and OS. A two-sided P < 0.05 was considered statistically significant.

## Results

### Patient characteristics

**Fig. 1:**
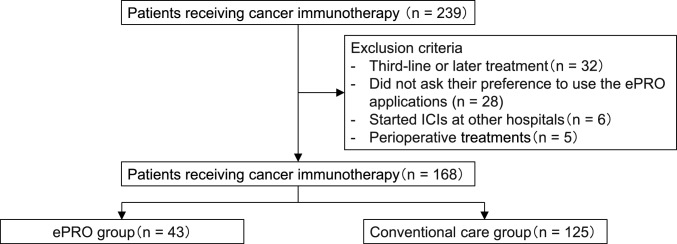
Flowchart for inclusion of patients

Among the 239 patients treated with ICIs at Komatsu Municipal Hospital, 71 were excluded for the following reasons: receipt of ICIs as third-line or later treatment (n = 32), lack of inquiry regarding their preference to use ePRO applications (n = 28), initiation of ICIs at other hospitals (n = 6), and receipt of ICIs as perioperative treatment (n = 5) (Fig. 1). A total of 168 patients were included in the analysis, of whom 43 (26%) opted to use ePROs (ePRO group), while the remaining 125 (74%) declined ePRO use (conventional care group). Reasons for declining ePRO use included not owning a smartphone (n = 57), lack of confidence in using the app (n = 33), perceiving it as unnecessary (n = 14), finding it bothersome (n = 11), or other reasons (n = 10). The median follow-up duration was 23.0 months (IQR: 7.5–26.6 months). Baseline patient characteristics are summarized in Table 1. The median age was 74 years (IQR: 69–79) and was significantly lower in the ePRO group than in the conventional care group (69 vs. 75 years, P < 0.001). Smartphone ownership was significantly higher in the ePRO group than in the conventional care group (100% vs. 54%, P < 0.001).

**Table 1:** Baseline characteristics of patients

	ePRO group (n = 43)	Conventional care group (n = 125)	P value
Age (years), median (IQR)	69 (65–74)	75 (71–79)	< 0.001
Sex, n (%)			0.735
Male	33 (77%)	99 (79%)	
Female	10 (23%)	26 (21%)	
ECOG PS, n (%)			0.749
0–1	37 (86%)	105 (84%)	
≥ 2	6 (14%)	20 (16%)	
Type of cancer, n (%)			0.100
Non–small cell lung cancer	28 (65%)	97 (78%)	
Small cell lung cancer	5 (12%)	10 (8%)	
Hepatocellular carcinoma	2 (5%)	5 (4%)	
Head and neck cancer	2 (5%)	3 (2%)	
Others	6 (14%)	10 (8%)	
Body surface area (m^2^), median (IQR)	1.63 (1.50–1.73)	1.59 (1.50–1.71)	0.663
NLR			
< 5	27 (63%)	86 (69%)	0.469
≥ 5	16 (37%)	39 (31%)	
Number of prior regimens [n (%)]			0.506
0	33 (77%)	90 (72%)	
1	10 (23%)	35 (28%)	
Regimen, n (%)			0.193
PD-1 + chemotherapy	15 (35%)	53 (42%)	
PD-1	13 (30%)	43 (34%)	
PD-1 + CTLA-4 ± chemotherapy	15 (35%)	29 (23%)	
Smartphone users, n (%)			< 0.001
Yes	43 (100%)	68 (54%)	
No	0 (0%)	57 (46%)	
Type of application, n (%)			–
Welby My Carte ONC^®^	34 (79%)	–	
MedicalCare Station^®^	5 (12%)	–	
3H P-Guardian^®^	4 (9%)	–	

*IQR* interquartile range, *ePRO* electronic patient-reported outcomes, *ECOG PS* Eastern Cooperative Oncology Group performance status, *NLR* neutrophil to lymphocyte ratio, *PD-1* programmed cell death protein 1 (PD-1) inhibitor or programmed death-ligand 1 inhibitor, *CTLA-4* cytotoxic T-lymphocyte antigen

### Safety

ICI-based chemotherapy–related adverse events observed in ≥ 5% of patients are presented in Table 2. The incidence of any adverse events and irAEs was similar between the ePRO and conventional care groups (88% vs. 86%, P = 0.741 and 56% vs. 56%, P = 0.983, respectively). Additionally, the incidence of grade ≥ 3 adverse events did not significantly differ between the ePRO and conventional care groups (33% vs. 45%, P = 0.160). However, the incidence of grade ≥ 3 irAEs was significantly lower in the ePRO group than in the conventional care group (7% vs. 21%; P = 0.039). Notably, of the nine patients with severe pneumonitis in the conventional care group, one patient died due to grade 5 pneumonitis, whereas no treatment-related deaths occurred in the ePRO group.

**Table 2:** Treatment-related adverse events reported in at least 5% of patients

	ePRO group (n = 43)		Conventional care group (n = 125)		Any grade	Grade ≥ 3	
	Any grade	Grade ≥ 3	Any grade	Grade ≥ 3	P value	P value	
Adverse events (%)							
Any event	38 (88%)	14 (33%)	108 (86%)	56 (45%)	NS	0.160	
Anorexia	13 (38%)	2 (6%)	43 (42%)	12 (12%)	NS	NS	
Anemia	13 (38%)	0 (0%)	23 (22%)	7 (7%)	NS	NS	
Constipation	12 (35%)	1 (3%)	19 (18%)	1 (1%)	0.064	NS	
Neutropenia	11 (32%)	7 (21%)	33 (32%)	22 (21%)	NS	NS	
Rash	11 (32%)	0 (0%)	35 (34%)	3 (3%)	NS	NS	
Malaise	9 (26%)	0 (0%)	41 (40%)	5 (5%)	NS	NS	
Pneumonia	8 (24%)	1 (3%)	19 (18%)	10 (10%)	NS	NS	
Fever	8 (24%)	0 (0%)	7 (7%)	0 (0%)	0.001	NS	
Leukopenia	7 (21%)	3 (9%)	18 (17%)	12 (12%)	NS	NS	
AST/ALT elevation	6 (18%)	2 (6%)	15 (15%)	5 (5%)	NS	NS	
Thrombocytopenia	6 (18%)	1 (3%)	22 (21%)	9 (9%)	NS	NS	
Alopecia	6 (18%)	0 (0%)	15 (15%)	0 (0%)	NS	NS	
Creatinine increased	5 (15%)	1 (3%)	13 (13%)	4 (4%)	NS	NS	
Mucositis oral	5 (15%)	0 (0%)	5 (5%)	1 (1%)	0.068	NS	
Dysgeusia	5 (15%)	0 (0%)	4 (4%)	0 (0%)	0.034	NS	
Peripheral neuropathy	4 (12%)	0 (0%)	23 (22%)	3 (3%)	NS	NS	
Diarrhea	3 (9%)	0 (0%)	21 (20%)	3 (3%)	NS	NS	
Febrile neutropenia	2 (6%)	2 (6%)	0 (0%)	0 (0%)	0.015	0.015	
Hypothyroidism	2 (6%)	0 (0%)	9 (9%)	1 (1%)	NS	NS	
Hemorrhage	2 (6%)	0 (0%)	3 (3%)	0 (0%)	NS	NS	
Adrenal hypofunction	2 (6%)	0 (0%)	4 (4%)	0 (0%)	NS	NS	
Inflammatory arthritis muscular pain	1 (3%)	0 (0%)	9 (9%)	1 (1%)	NS	NS	
Proteinuria	1 (3%)	1 (3%)	5 (5%)	5 (5%)	NS	NS	
Immune-related adverse events (%)							
Any event	24 (56%)	3 (7%)	70 (56%)	26 (21%)	NS	0.039	
Rash	9 (26%)	0 (0%)	31 (30%)	3 (3%)	NS	NS	
Pneumonia	7 (21%)	1 (3%)	15 (15%)	9 (9%)	NS	NS	
Fever	5 (15%)	0 (0%)	5 (5%)	0 (0%)	0.068	NS	
Creatinine increased	4 (12%)	1 (3%)	8 (8%)	3 (3%)	NS	NS	
AST/ALT elevation	3 (9%)	1 (3%)	8 (8%)	3 (3%)	NS	NS	
Hypothyroidism	2 (8%)	0 (0%)	9 (9%)	1 (1%)	NS	NS	
Adrenal hypofunction	2 (6%)	0 (0%)	4 (4%)	0 (0%)	NS		NS
Diarrhea	0 (0%)	0 (0%)	10 (10%)	2 (2%)	0.056		NS
Malaise	1 (3%)	0 (0%)	5 (5%)	0 (0%)	NS		NS

*ePRO* electronic patient-reported outcome, *AST* aspartate aminotransaminase, *ALT* alanine aminotransferase, *NS* not significant

### Emergency visits and hospitalization

The proportion of patients with emergency visits was significantly lower in the ePRO group than in the conventional care group (23% vs. 59%, P < 0.001; Table 3). A similar trend was observed for emergency hospitalizations (16% vs. 34%, P = 0.031). In univariate analyses, ePRO use (OR 0.21, 95% CI 0.10–0.46, P < 0.001) and having NSCLC (OR 3.03, 95% CI 1.45–6.37, P = 0.003) were significantly associated with emergency visits among patients receiving ICIs (Table 4). Similarly, the risk of emergency hospitalizations was significantly associated with ePRO use (OR 0.38, 95% CI 0.16–0.94, P = 0.031), PS ≥ 2 (OR 2.94, 95% CI 1.25–6.94, P = 0.011), and NLR ≥ 5 (OR 2.12, 95% CI 1.06–4.24, P = 0.031). In the subsequent multivariate analysis, ePRO use (OR 0.22, 95% CI 0.10–0.50, P < 0.001) and having NSCLC (OR 2.81, 95% CI 1.30–6.08, P = 0.009) remained significantly associated with emergency visits. Similarly, the risk of emergency hospitalizations was also significantly associated with the use of ePROs (OR 0.36, 95% CI 0.14–0.89, P = 0.027) and PS ≥ 2 (OR 2.62, 95% CI 1.07–6.41, P = 0.034).

**Table 3:** Emergency visits and hospitalizations

	ePRO group (n = 43)	Conventional care group (n = 125)	P value
Emergency visits, n (%)			
Yes	10 (23%)	74 (59%)	< 0.001
No	33 (77%)	51 (41%)	
Emergency hospitalizations, n (%)			
Yes	7 (16%)	42 (34%)	0.031
No	36 (84%)	83 (66%)	

*ePRO* electronic patient-reported outcome

**Table 4:** Univariate and multivariate analyses of risk factors for emergency visit and hospitalization

Variables	Emergency visits				Emergency hospitalizations			
	Univariate analysis		Multivariate analysis		Univariate analysis		Multivariate analysis	
	OR (95% CI)	P value	OR (95% CI)	P value	OR (95% CI)	P value	OR (95% CI)	P value
Use of ePROs	0.21 (0.10–0.46)	< 0.001	0.22 (0.10–0.50)	< 0.001	0.38 (0.16–0.94)	0.031	0.36 (0.14–0.89)	0.027
NSCLC	3.03 (1.45–6.37)	0.003	2.81 (1.30–6.08)	0.009	1.24 (0.59–2.62)	0.571	N/A	–
ECOG PS ≥ 2	1.44 (0.62–3.36)	0.394	N/A	–	2.94 (1.25–6.94)	0.011	2.62 (1.07–6.41)	0.034
NLR ≥ 5	1.31 (0.69–2.50)	0.411	N/A	–	2.12 (1.06–4.24)	0.031	2.06 (1.00–4.26)	0.051
Age ≥ 75 years	1.71 (0.92–3.17)	0.087	N/A	–	1.54 (0.79–3.01)	0.204	N/A	–
Previous chemotherapies	1.21 (0.60–2.42)	0.115	N/A	–	0.47 (0.20–1.10)	0.077	N/A	–

*OR* odds ratio, *CI* confidence interval, *ePROs* electronic patient-reported outcome, *NSCLC* non–small cell lung cancer, *ECOG PS* Eastern Cooperative Oncology Group performance status, *NLR* neutrophil-to-lymphocyte ratio, *N/A* not applicable

### Efficacy

**Fig. 2:**
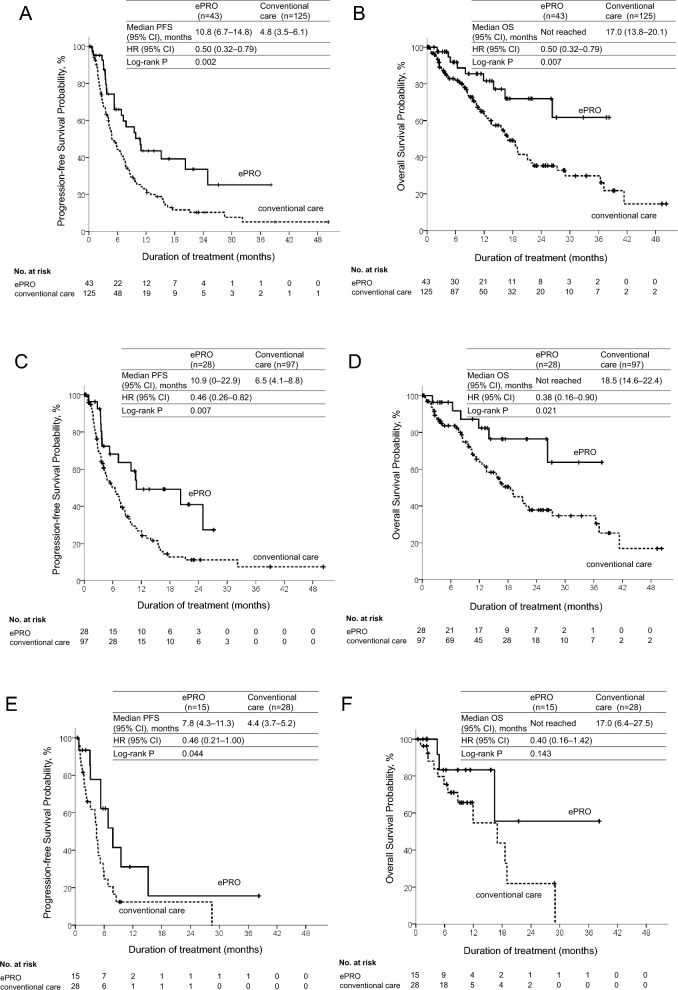

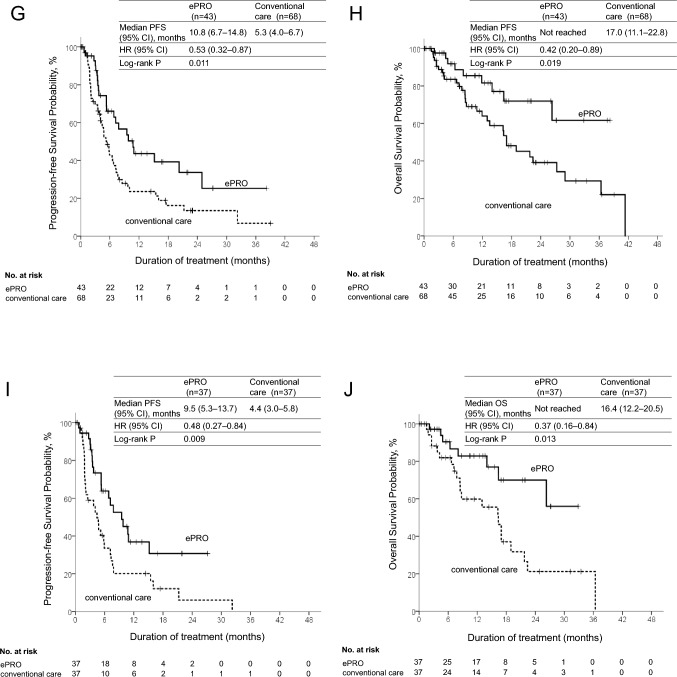
Progression-free survival (PFS) and overall survival (OS) outcomes in the electronic patient-reported outcome (ePRO) group versus conventional care group during immune checkpoint inhibitor-based therapy. Kaplan–Meier curves for PFS and OS of all patients **A**, **B**, only those with non-small cell lung cancer (NSCLC) **C**, **D**, excluding those with NSCLC **E**, **F**, smartphone-owning patients **G**, **H**, and the propensity score-matched cohort **I**, **J**. Median survival values with 95% confidence intervals (CI), hazard ratios (HR) with 95% CI, and log-rank P values are shown in each panel. The number of patients at risk is indicated below each curve

At the last follow-up, 72 patients (43%) had died, 9 in the ePRO group and 63 in the conventional care group. The median PFS and OS were 10.8 months (95% CI 6.7–14.8) versus 4.8 months (95% CI 3.5–6.1), and not reached versus 17.0 months (95% CI 13.8–20.1), respectively (Fig. 2A and B). Both PFS and OS were significantly longer in the ePRO group than in the conventional care group (HR 0.50, 95% CI 0.32–0.79, P = 0.003 and HR 0.40, 95% CI 0.20–0.80, P = 0.010, respectively). In the NSCLC subgroup, ePRO use was similarly associated with improved PFS and OS (HR 0.46, 95% CI 0.26–0.82, P = 0.007 and HR 0.38, 95% CI 0.16–0.90, P = 0.021; Fig. 2C and D). In the non-NSCLC subgroup, PFS was also significantly prolonged (HR 0.46, 95% CI 0.21–1.00; P = 0.044), whereas OS did not reach significance (HR 0.40, 95% CI 0.16–1.42; P = 0.143; Fig. 2E and F). In smartphone-owning patients, similar benefits were observed for PFS and OS (HR 0.53, 95% CI 0.32–0.87, P = 0.013 and HR 0.42, 95% CI 0.20–0.89, P = 0.023; Fig. 2G and H). Multivariate analysis confirmed that ePRO use was independently associated with PFS and OS (HR 0.45, 95% CI 0.28–0.71, P < 0.001 and HR 0.38, 95% CI 0.19–0.77, P = 0.007, respectively; Table 5). The objective response and disease control rates were significantly higher in the ePRO group than in the conventional care group (40% vs. 22%, P = 0.038 and 79% vs. 54%, P = 0.004, respectively; Supplementary Table S1). After propensity score matching (n = 37 per group), the survival benefit remained significant (PFS: 9.5 vs. 4.4 months, HR 0.48, 95% CI 0.27–0.84, P = 0.011; OS: not reached vs. 16.4 months, HR 0.37, 95% CI 0.16–0.84, P = 0.017; Fig. 2I and J).

**Table 5:** Univariate and multivariate analyses of association between clinical characteristics and progression-free survival, or overall survival in patients who received ICI-based chemotherapy

Variables	Progression-free survival				Overall survival			
	Univariate analysis		Multivariate analysis		Univariate analysis		Multivariate analysis	
	OR (95% CI)	P value	HR (95% CI)	P value	OR (95% CI)	P value	HR (95% CI)	P value
Use of ePROs	0.50 (0.32–0.79)	0.003	0.45 (0.28–0.71)	0.001	0.40 (0.20–0.80)	0.010	0.38 (0.19–0.77)	0.007
NSCLC	1.42 (0.94–2.13)	0.095	1.70 (1.12–2.59)	0.013	1.28 (0.73–2.24)	0.394	N/A	–
ECOG PS ≥ 2	1.48 (0.93–2.36)	0.097	N/A	–	2.15 (1.21–3.83)	0.009	2.28 (1.28–4.05)	0.005
NLR ≥ 5	1.28 (0.88–1.87)	0.199	1.45 (0.99–2.12)	0.058	1.32 (0.81–2.16)	0.268	N/A	–
Age ≥ 75 years	0.98 (0.68–1.40)	0.894	N/A	–	1.13 (0.71–1.81)	0.597	N/A	–
Previous chemotherapies	1.04 (0.69–1.56)	0.859	N/A	–	1.13 (0.67–1.91)	0.654	N/A	–

*HR* hazard ratio, *CI* confidence interval, *ePROs* electronic patient-reported outcomes, *NSCLC* non–small cell lung cancer, *ECOG PS* Eastern Cooperative Oncology Group performance status, *NLR* neutrophil-to-lymphocyte ratio, *N/A* not applicable

## Discussion

In this retrospective study, we found that ePRO use in patients with advanced cancer receiving ICI-based chemotherapy was associated with reduced rates of severe irAEs, emergency visits, and hospitalizations, as well as improvements in PFS and OS. To our knowledge, this study is the first to investigate the utility of ePROs in patients treated with ICIs in a real-world clinical setting. ePRO-based symptom monitoring facilitates timely patient–clinician communication, leading to earlier management and improved symptom control [15]. In the conventional care group, symptom information was obtained primarily at scheduled clinic visits; between visits, symptom changes—including irAE onset or worsening—could not be detected unless patients independently sought medical attention. In contrast, the ePRO system provided continuous patient-reported symptom data between visits, enabling more informed assessment. This may have allowed healthcare providers to identify and respond to symptom changes more effectively than conventional visit-based assessment alone. In this study, although the overall incidence of irAEs was similar between the ePRO and the conventional care groups, the incidence of grade ≥ 3 irAEs was significantly lower in the ePRO group. These results suggest that symptom monitoring using ePROs during ICI-based chemotherapy enables early detection and management of irAEs before they progress to severe stages. Notably, grade ≥ 3 rash (0% vs. 3%; any grade: 26% vs. 30%) and grade ≥ 3 pneumonitis (3% vs. 9%; any grade: 21% vs. 15%) were less frequent in the ePRO group. One patient in the conventional care group died from grade 5 pneumonitis, whereas no treatment-related deaths occurred in the ePRO group. A non-significant reduction in grade ≥ 3 anorexia (6% vs. 12%) suggests broader benefits of ePRO monitoring beyond irAEs. These reductions in severe adverse events may have contributed to lower rates of emergency visits and hospitalizations in the ePRO group. Multivariate analysis confirmed that ePRO use remained significantly associated with fewer emergency visits and hospitalizations. Consistent with prior randomized and real-world studies showing reduced emergency visits and hospitalizations with ePRO use [16, 17], a randomized clinical trial specifically in patients receiving immunotherapy similarly demonstrated reductions in serious irAEs and emergency department visits [9]. Notably, approximately 50% of cancer-related emergency visits have been considered potentially preventable through PRO-based monitoring [18]. Given that approximately 63% of such visits result in hospitalization [19], these findings collectively support the role of ePROs in reducing both outcomes via early adverse event detection. Our findings suggest that ePRO use may contribute to reduced healthcare utilization. Importantly, the ePRO group demonstrated improvement in the PFS and OS. Although the proportion of NSCLC (74%) in the study population and younger patient age in the ePRO group may have influenced the findings, the association with improved survival persisted despite these baseline imbalances. Subgroup analyses by cancer type consistently showed improved PFS and OS with ePRO use in both NSCLC and non-NSCLC patients. Multivariate analysis further confirmed this survival advantage (PFS: HR 0.45; OS: HR 0.38), as did propensity score matching (n = 37 per group; PFS: HR 0.48; OS HR 0.37). As per previous randomized controlled trials, ePRO-based symptom monitoring in patients with cancer is associated with prolonged survival compared with conventional care [20, 21]. Another randomized controlled trial reported improved mortality with PRO-based symptom monitoring in patients with advanced cancer receiving ICI-based chemotherapy; however, the difference was not statistically significant (HR 0.38; P = 0.28) [9]. Low-grade (grade 1–2) irAEs are associated with favorable ICI efficacy, whereas grade ≥ 3 irAEs are associated with poorer survival outcomes [22, 23]. In the present study, although the overall incidence of irAEs was similar between the ePRO and conventional care groups, the incidence of grade ≥ 3 irAEs was significantly lower in the ePRO group. This reduction may partly explain the improved PFS and OS. As grade ≥ 3 irAEs often require treatment interruption or high-dose corticosteroids that may attenuate ICI efficacy, early detection and management of irAEs may have helped maintain treatment continuity. Furthermore, ePRO-based monitoring may have enhanced patient engagement and self-management, thereby improving treatment adherence. Prior studies have shown longer treatment duration with ePRO use in patients receiving chemotherapy [20] and lower discontinuation rates in those receiving ICI-based chemotherapy [9], suggesting that ePRO-based monitoring may help patients remain on treatment, contributing to improved survival. The consistent findings in smartphone-owning patients further suggest that the survival benefit was not explained solely by differences in digital literacy.

This study has a few notable limitations. First, this was a single-center, retrospective analysis with a small sample size, which may limit the generalizability of our findings. Second, because of the nonrandomized design and the nature of ePRO implementation, there were imbalances in baseline patient characteristics between the two groups, most notably in age and smartphone ownership. Device-related selection bias, whereby younger smartphone-owning patients were more likely to adopt ePROs, may have confounded the survival outcomes. However, the survival benefit remained significant after multivariable adjustment and additionally after propensity score matching, supporting the robustness of our findings. Nevertheless, residual confounding due to unmeasured factors cannot be excluded, and the generalizability of these findings to older or digitally inexperienced patients may be limited. Third, the use of three ePRO platforms and variability in reporting frequency may have introduced heterogeneity in monitoring intensity. Although common procedures—including CTCAE-based severity grading, routine pharmacist review, and predefined alert-based responses—were applied consistently across platforms, differences in interface design and patient adherence should be considered. The standardized monitoring workflow, rather than the specific platform, may be a key factor underlying the observed outcomes; however, this warrants validation in future prospective multicenter studies.

In conclusion, this real-world study investigated the potential benefits of ePRO-based symptom monitoring in patients with advanced cancer receiving immunotherapy. Our findings suggest that the ePRO follow-up model may contribute to improved safety outcomes and OS in patients receiving immunotherapy. However, these findings warrant validation in larger prospective studies. Future research may further optimize patient care in immunotherapy.

## Supplementary Information

Below is the link to the electronic supplementary material.

Supplementary file1 (PDF 134 KB)

## Declarations

### Data availability

The data that support the findings of this study contain personal information and are therefore not publicly available. De-identified data may be provided by the corresponding author upon reasonable request and subject to institutional approval.
