# Supplementary material for: Effectiveness of electronic patient-reported outcomes in patients with cancer undergoing immunotherapy: a real-world retrospective study
Source: Int J Clin Oncol. 2026 Apr 13;31(7):1215–25. doi: 10.1007/s10147-026-03032-z (PMC13303399; doi:10.1007/s10147-026-03032-z)
Supplement: Supplementary file 1 — Supplementary file1 (PDF 134 KB) [file 10147_2026_3032_MOESM1_ESM.pdf]

Effectiveness of Electronic Patient-Reported Outcomes in Patients with Cancer Undergoing Immunotherapy: A Real-World Retrospective Study

Hideyuki Katsura[1,*], Yukio Suga[2], Shinji Kimoto[3], Reiko Ando Makihara[4], Hiroshi Ota[1], Hitomi Toi[1], Naoko Takata[1], Hiroaki Ikesue[5]

[1] Department of Pharmacy, Komatsu Municipal Hospital, HO-60, Mukai Moto-ori-machi, Komatsu, Ishikawa 923-8560, Japan.

[2] Department of Clinical Drug Informatics, Faculty of Pharmacy, Institute of Medical, Pharmaceutical & Health Science, Kanazawa University, 13-1, Takaramachi, Kanazawa, Ishikawa 920-8641, Japan.

[3] Department of Pharmacy, Takeda General Hospital; 3-27 Yamaga-machi, Aizuwakamatsu, Fukushima, 965-8585, Japan.

[4] Department of Pharmacy, National Cancer Center Hospital, 5-1-1 Tsukiji, Chuo-ku, Tokyo 104-0045, Japan.

[5] Department of Pharmacy, Nagoya University Hospital; 65, Tsurumai-cho, Showa-ku, Nagoya, Aichi 466-8560, Japan.

***Corresponding Author:** Hideyuki Katsura

Department of Pharmacy, Komatsu Municipal Hospital, HO-60, Mukai Moto-ori-machi, Komatsu, Ishikawa 923-8560, Japan.

ORCID: 0009-0007-1930-6236

Tel: +81-76-122-7111, Fax: +81-76-124-0792

E-mail: hideyuki_katsura@yahoo.co.jp

Supplementary Information

Supplementary Table S1. Therapeutic response in patients treated with ICI-based chemotherapy

Best overall response, n (%)	ePRO group (n = 43)	Conventional care group (n = 125)
Complete response	0 (0%)	1 (1%)
Partial response	17 (40%)	28 (22%)
Stable disease	17 (40%)	39 (31%)
Progressive disease	3 (7%)	47 (39%)
Unknown	6 (14%)	10 (8%)

ePRO, electronic patient-reported outcomes. Response rates (40% vs. 22%, $p = 0.038$) and disease control rates (79% vs. 54%, $p = 0.004$) were significantly higher in the ePRO group than in the conventional care group.
